# Reducing inequalities in health and access to health care in a rural Indian community: an India-Canada collaborative action research project

**DOI:** 10.1186/1472-698X-11-S2-S3

**Published:** 2011-11-08

**Authors:** Slim Haddad, Delampady Narayana, KS Mohindra

**Affiliations:** 1 Centre de recherche du Centre Hospitalier de l’Université de Montreal (CHUM), Université de Montréal, 3850 rue Saint-Urbain, Montréal, Québec, Canada; 2 Centre for Development Studies, Prasanth Nagar, Ulloor, Thiruvananthapuram. 695 011, Kerala, India; 3 Institute of Population Health, University of Ottawa, Ottawa, Canada

## Abstract

**Background:**

Inadequate public action in vulnerable communities is a major constraint for the health of poor and marginalized groups in low and middle-income countries (LMICs). The south Indian state of Kerala, known for relatively equitable provision of public resources, is no exception to the marginalization of vulnerable communities. In Kerala, women’s lives are constrained by gender-based inequalities and certain indigenous groups are marginalized such that their health and welfare lag behind other social groups.

**The research:**

The goal of this socially-engaged, action-research initiative was to reduce social inequalities in access to health care in a rural community. Specific objectives were: 1) design and implement a community-based health insurance scheme to reduce financial barriers to health care, 2) strengthen local governance in monitoring and evidence-based decision-making, and 3) develop an evidence base for appropriate health interventions.

**Results and outcomes:**

Health and social inequities have been masked by Kerala’s overall progress. Key findings illustrated large inequalities between different social groups. Particularly disadvantaged are lower-caste women and Paniyas (a marginalized indigenous group), for whom inequalities exist across education, employment status, landholdings, and health. The most vulnerable populations are the least likely to receive state support, which has broader implications for the entire country. A community based health solidarity scheme (SNEHA), under the leadership of local women, was developed and implemented yielding some benefits to health equity in the community—although inclusion of the Paniyas has been a challenge.

**The partnership:**

The Canadian-Indian action research team has worked collaboratively for over a decade. An initial focus on surveys and data analysis has transformed into a focus on socially engaged, participatory action research.

**Challenges and successes:**

Adapting to unanticipated external forces, maintaining a strong team in the rural village, retaining human resources capable of analyzing the data, and encouraging Paniya participation in the health insurance scheme were challenges. Successes were at least partially enabled by the length of the funding (this was a two-phase project over an eight year period).

## Background

Inadequate public action in vulnerable communities is a major constraint for the health of poor and marginalized groups in low and middle-income countries (LMICs) and can manifest in various ways. First, lack of social protection compounded by financial barriers can lead to exclusion from health care and impoverishment, trapping families in a cycle of poverty and ill health [1]. Second, diminished opportunities for good health can lead to poor health outcomes [2]. Finally, a lack of relevant data contributes to poor planning for addressing the felt needs of populations. The south Indian state of Kerala, known for relatively equitable provision of public resources [3], is no exception to the marginalization of vulnerable communities.

Kerala is known for achieving impressive health outcomes at modest incomes. Women in Kerala fare relatively well, which is reflected in their high literacy and low mortality rates, despite severe gender discrimination in the country at large [4]. Kerala’s success has been attributed largely to historical particularities and progressive public policies implemented by successive governments [5]. However, a number of emerging challenges in this society threaten its health equity. First, there has been a decline in public health services, precipitating a shift towards the use of private health services even among the poor [6]. Rising health care costs are impoverishing households and leading poor and marginalized groups to avoid seeking care. Second, despite Kerala’s embarking on an ambitious decentralization movement to promote equity-oriented policies, local governments lack proper tools and systematic approaches to monitor needs and access to services. Finally, Kerala’s “egalitarian” reputation is somewhat misplaced, as women’s lives are still constrained by gender-based inequalities (e.g. domestic violence), [7,8] and certain indigenous groups (known as Scheduled Tribes, or ST) are marginalized, such that their levels of health and welfare lag behind other social groups [9]. Despite more than 50 years of affirmative action policies in India, STs have received inadequate attention from decision-makers and researchers [10,11].

## The research

The overarching goal of this research initiative was to reduce social inequalities in access to health care in a rural community (Kottathara Panchayat). Specifically, our objectives were to: 1) design and implement a community-based health insurance (CBHI) scheme to reduce financial barriers to health care, especially among the poor, 2) strengthen local governance capacities in monitoring and promote a culture of evidence-based decision-making, and 3) develop an evidence base for appropriate interventions to improve the health of the most vulnerable Paniya tribe (a previously enslaved ST) using appropriate ethical and methodological approaches.

**Figure 1:**
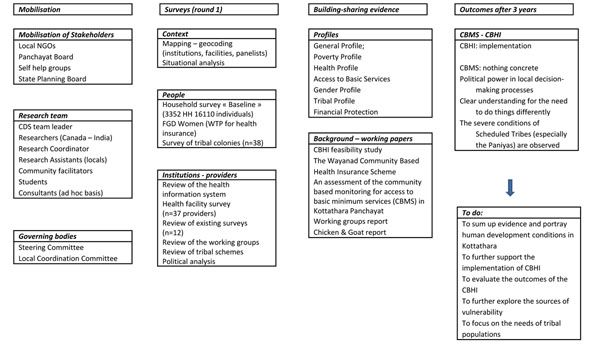
**Phase I activities (2002-2005)**. Phase I activities included: 1) mobilizing partners by holding individual and community-wide meetings; 2) implementing surveys; 3) analyzing data, preparing detailed but easy to read statistical profiles of Kottathara, and presenting findings to the community and scientific audience; and 4) designing the CBHI.

**Figure 2:**
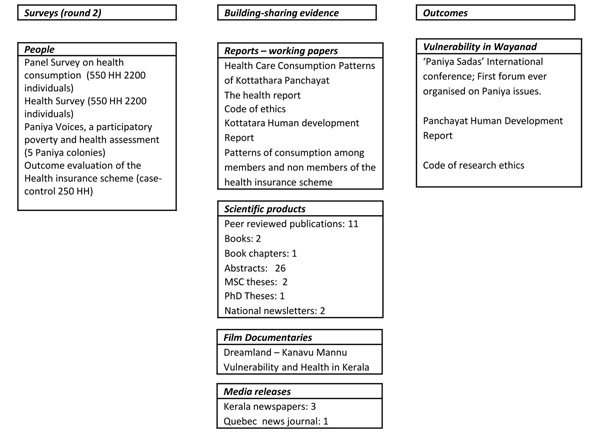
**Phase II activities (2006-2010).** Phase II activities included: 1) implementing the CBHI and later supporting it as an autonomous body; 2) preparing and releasing the Kottathara Human Development Report; and 3) undertaking “Paniya Voices”, a participatory study with the Paniyas that culminated in a large forum in 2010.

Our aim was to balance scientific rigour with social engagement to improve access to health care and reduce social inequalities in health. We pursued the research initiative (in which the field work was led by the Indian partner at the Centre for Development Studies) in two phases. Phase I activities (2002–2005) included: 1) mobilizing partners by holding individual and community-wide meetings; 2) implementing surveys; 3) analyzing data, preparing detailed but easy to read statistical profiles of Kottathara, and presenting findings to the community and scientific audience; and 4) designing the CBHI (see Figure 1). Phase II activities (2006–2010) included: 1) implementing the CBHI and later supporting it as an autonomous body; 2) preparing and releasing the Kottathara Human Development Report; and 3) undertaking “Paniya Voices”, a participatory study with the Paniyas that culminated in a large forum in 2010 (see Figure 2).

We adopted a common process for each objective: 1) documenting felt population needs; 2) implementing an intervention that would provide some form of social protection or community empowerment; and 3) producing sustainable outcomes that would endure beyond the project. Below, we examine the process in more detail for each objective.

The idea of a CBHI arose in discussions with women’s self-help groups (a form of microcredit). The women, who were extremely knowledgeable about basic banking operations and credit activities, observed that they often had to use their loans to meet their families’ health care costs, which reduced their opportunities to invest in productive activities [12]. Analysis of the data collected in surveys confirmed their observation. Women in self-help groups became our primary partners in developing a suitable design for a CBHI that could ultimately become an autonomous body.

As part of the Community Based Monitoring System, we held a series of workshops with local government decision-makers to assess their capacities and their preferences for working towards data-based decision-making. The participants underscored the difficulty in selecting indicators for monitoring. The research team therefore prepared a presentation package using a selection of 17 different indicators, which resulted in a higher level of discussions on technical and practical issues. The interest in the local-level data collected by the project later led to the development of the Kottathara Human Development Report (see Table 1 for a fuller description), which was then disseminated at the local and state levels, both as a source of information and a potential tool for other panchayats.

**Table 1:** Kottathara Human Development Report

The Kerala State Planning Board (KSPB) invited the Centre for Development Studies to prepare a local Human Development Report (at the Gram Panchayat level) as a follow-up of the Kerala Human Development Report 2005 [17]. The KoHDR seeks to consolidate the momentum gained in preparing the KHDR and strengthen the State planning process to focus on human development concerns. It presents an analysis of the achievements and the problems facing Kottathara Gram Panchayat in poverty reduction, health and education, and empowerment of the socially disadvantaged. This approach, to our knowledge the first of its kind to be undertaken in India, was made possible thanks to in-depth knowledge developed by the research team, including the numerous project profiles and databases, which were extensively used in preparing the Kottathara Human Development Report.

With regard to marginalized STs, analysis of the data showed high levels of health needs among the Paniyas and large disparities between the Paniyas and the other tribes, on one hand, and between the tribes and other social groups, on the other. However, the standardized surveys we implemented were not sufficiently sensitive to provide any understanding of the underlying sources of vulnerability, and there was also evidence that the Paniyas did not use the public services and programs available to them. This led to the study “Paniya Voices”, in which we developed a new approach, which we called “participatory poverty and health assessments”, using additional ethical strategies (e.g. the Ethical Code of Research) [13]. This study provided a rich source of information, and its approach empowered the participating colonies. Drawing on the momentum created by this study and on the resources of a network of social activists and the Paniya community, a forum was held (“Paniya Sadas”) that brought together decision-makers, researchers and citizens to discuss possible solutions for improving the lives of the Paniyas. At this groundbreaking event, a 300-page document on the Paniyas’ health, education and economic deprivation was discussed for three days, with about 60 Paniyas participating.

## Results and outcomes

### Key findings and analysis of issues

The key findings are summarized in Tables 2 and 3. Our initiative produced two main categories of outcomes: methodological advances and scientific knowledge for action. The methodological advances included two main contributions. The first concerned health indicators. Typically in LMICs questionnaires are implemented using self-reported health indicators. Our analyses demonstrated the limitations of this approach. Due to what Amartya Sen has labelled a perception bias [14], these indicators can bias the results. Specifically, we found that the Paniyas, the poorest and most marginalized group, reported levels of health comparable to the highest-ranked social group, although they had the worst measures of objective health [15]. We therefore concluded that undertaking research on social inequalities in health among these vulnerable populations calls for the use of multiple indicators.

**Table 2:** Key findings from Phase I

Social inequalities in health **■** Caste and socioeconomic inequalities in women’s health were observed: 1) women from lower castes reported a higher prevalence of poor health than women from high castes; 2) socioeconomic inequalities in health existed regardless of the indicator used (education, women’s employment status, or household landholdings; and 3) multilevel models indicated that among women with low caste affiliations, the influence of socioeconomic indicators led to a “magnifying” effect, whereas among women with high caste affiliations, a “buffering” effect was found [15].
**Health of Paniyas** **■** Compared to non-Paniyas, Paniyas reported: 1) the highest rates of poverty, 2) the lowest education levels; 3) the lowest utilization rates of health care facilities; 4) the highest household expenditure on alcohol; 5) the worst access to safe drinking water and sanitation facilities, and 6) the poorest housing conditions [project profiles].

**Population health interventions** **■** Poor women who participated in a self-help group (SHG) were: 1) less likely to face exclusion from health care compared to poor women who did not participate in a SHG, and 2) less likely to report emotional stress and poor life than poor women who did not participate in a SHG if the woman had been a member of a SHG for at least two years [12]. **■** SNEHA was successfully launched and the community based health insurance (CBHI) became operational in July 2005.

**Table 3:** Key findings from Phase II

Social inequalities in health **•** There is a large health divide between social groups. The category of SC/ST (targeted by positive discrimination) is heterogeneous: one ST group, the Paniyas, are particularly disadvantaged. The divide between ST and non-ST populations persists even after controlling for poverty and education. These results illustrate that STs’ vulnerability in the social structure goes well beyond material deprivation [18].
**Health of Paniyas** **■** Paniyas have high levels of health needs; their prevalences of underweight, anaemia, and goitre are 60%, 15%, and 11% respectively [18]. Inequalities in health exist across generations. Young adults are particularly disadvantaged with respect to hypertension. Paniyas reported that they are caught in multiple “vulnerability traps”, that is, they view their situation as vicious cycles from which it is difficult to break away [13;19]. **■** Alcohol is viewed as a problem among the Paniyas who reported that: 1) its consumption is increasing, notably among younger men; 2) it is easily available in licensed shops and is produced illicitly in some colonies; 3) it is used to attract Paniyas for work by some local employers; and 4) it is associated with a range of social and economic consequences that are rooted in historical oppression and social discrimination [16].

**Population health interventions** **■** Women office bearers of SNEHA have proven themselves capable of decisionmaking and tackling challenges proactively (see, for instance, the change of insurance company). They have developed their negotiating power (see the negotiation with the insurance company that took over the scheme), have gained a profound knowledge of health insurance and of inclusion issues (see the revision of eligibility criteria for enrolling in the CBHI), have shown leadership and have overcome political pressure (SNEHA at present is not ruled by its governing body but by informal leadership). Today the CBHI is still functional with about 1,000 members.

Our second methodological contribution was our approach to the exploration of socially vulnerable and culturally distinct groups such as STs, which involved helping them to express their own voices and to get to the root of a number of sensitive issues, such as alcohol consumption [16]. Participatory approaches, such as those developed by our team, originated in the field of development but are insufficiently used by global health researchers. Our work paves the way for future developments in using participatory approaches with such populations.

Our initiative made three main contributions to scientific knowledge for future action. First, working closely with the community, we conducted an in-depth analysis of population health that revealed important health disparities among certain groups, such as the Paniyas, that have been masked by Kerala’s overall progress. Second, we noted that health vulnerability appears to be anchored in the social structure, and that more than 50 years of progressive policies have scarcely reduced it; this is troubling precisely because the context of the study is a so-called “egalitarian” society. Third, we observed major inefficiencies in the structure and implementation of policies targeting the STs, due mainly to the application of uniform policies to all STs, which are heterogeneous groups. The most vulnerable populations are the least likely to receive state support, thereby perpetuating their poverty and social marginalization. This has broader implications for India, since similar affirmative action is being pursued in other states and at the federal level.

### Contributions to global health research

Global health research has become progressively established as a specialized field. Research practices converge around epistemological and ethical principles that are now relatively well articulated and largely accepted by the global health research community. These principles can be classified into three main categories. The first subscribes to what Dowdy [20] has qualified as “positive” ethical obligations that seek to promote the social value of research. These obligations encompass: 1) pursuing scientific excellence [21-23]; 2) aligning research with the priorities of the communities and countries involved [24-27] to avoid scientific colonialism [28] and to promote research that is responsive to the needs of populations [29]; and 3) forming collaborative partnerships between researchers in the North and South, on one hand, and between researchers, decision-makers and communities, on the other [21-25]. Equitable partnerships are usually considered a key component of global health research practice in order to reduce the risk of scientific colonialism [28] and exploitation of communities and researchers in the South [22,30], as well as to distribute research benefits equitably [24,25].

The principles in the second category have intrinsic value while also facilitating the three obligations mentioned above. These include: 1) pursuing large, intersectoral and interdisciplinary research projects that address the determinants of health [26,31,32]; 2) engaging researchers to apply and integrate knowledge into practice [25,31,32]; 3) engaging institutions and researchers that favour developing research capacities, especially among partners in the South [21-23,25-27]; 4) engaging researchers as “progressive citizens” for development that will meet fundamental human rights [33], attain the Millennium Development Goals (MDGs) [26] and promote equity in health [23,34,35]; and 5) establishing programs and research collaborations that favour sustainable partnerships, strengthen local capacities and ensure that the final stages of the research process will further knowledge transfer [23].

The third category consists of ethical principles that are typically implemented to protect vulnerable populations [20,22,36]. According to Emmanuel and colleagues [22], these obligations include fair selection of study populations, favourable risk ratios, independent ethical review, informed consent, and respect for participants and communities.

In our research project, we made every attempt to apply all these principles. Our experience contributed to global health research in three key respects. First, our action research initiative revealed the inextricably complex mechanisms that determine the health of populations, underlining the importance of historical factors, social structure and the rights and opportunities of individuals and groups. This finding highlights the need for a truly multidisciplinary approach drawing on different epistemological traditions. Such an approach can help in understanding the mechanisms of a society such as Kerala and how its transformation contributes to reducing, maintaining, or exacerbating vulnerability in the health of its population and social groups.

Second, our model of socially engaged research gave a voice to groups such as Paniyas and women, who otherwise had little or no opportunity to be heard. Notably, the micro-interventions developed and implemented by our team succeeded solely on the basis of this engagement: the CBHI, in the case of poor women, and the Paniya Sadas, in the case of the STs. The researchers in this initiative thus adopted the role of actors for social change.

Third, in the “Paniya Voices” study, we developed and implemented an Ethical Code of Research [37], which formed the basis of the ethical and practical strategies used in the research. There have been increasing calls to strengthen ethical practices in global health research, especially to ensure that appropriate approaches are used with marginalized populations [38-40]. We implemented an ethical code that we had adapted from an earlier experience with indigenous populations in Quebec [41]; ethical codes are now considered best practice in undertaking indigenous health research in Canada [42]. To our knowledge this is the first time an ethical code has been implemented with an indigenous population in a LMIC.

## The partnership

The bonding of the Canada–India team began in Ottawa in 1997. The India team, while participating in an earlier eight-country study, had transcended its mandate and developed the data entry system, which it then installed in other countries. A commitment to academic rigour and a belief in something innovative brought together the team leaders of that study from Colombia, Thailand, Zimbabwe, India and the University of Montreal. Close friendships developed and common research interests were identified which later developed into research partnerships.

The partnership between the two principal investigators (SH, a physician and public health specialist, and DN, a development economist specializing in health systems) was founded on mutual respect, trust and transparency, with equal sharing of responsibility and acceptance of each other’s strengths and weaknesses. The overriding force was their keen interest in the subject and commitment to a cause. They took full responsibility for the behaviour of students and junior researchers as distinct work cultures came together in the project and mutual accommodations were made (food habits, language, etc). Further, there was complete transparency on all financial matters as money flowed directly from the IDRC to the participating institutions.

The initial focus on survey-based research and data analysis gradually transformed in the direction of understanding local governance, political analysis, marginalization, gender and empowerment. The Canadian PI’s strengths in health research and statistical rigour were combined with the Indian PI’s economics and development studies orientation. As junior researchers and students joined the team, and as the aspects of clinical research, nutrition, participatory research and study of indigenous populations were added, the project became a crucible of intense learning, sending a strong message to the Canadian team that the narrow boundaries of economics had to be transcended to understand social systems with diverse caste and religious identities.

## Challenges and successes

The initiative encountered four key challenges. First, there were a number of factors outside the project’s control that influenced the timing and nature of our activities. Examples include: a change in local government (between phase I and phase II) with a concomitant decrease in interest in evidence-based decision-making; a government proposal to provide health insurance for the poor, which was quickly retracted, thereby causing the community to mistrust the CBHI; and an uprising of tribal communities in the area, with the resultant tensions between tribal and nontribal communities.

Second, it was a challenge to maintain a strong local field team in a study site located in a remote area (a 12-hour train ride from the Centre for Development Studies), as most junior researchers were not interested in living there. Researchers spent significant time travelling to the study site. In addition, women who joined the project were often constrained by marriage and family obligations. With the exception of two team members, there was extensive turnover, necessitating constant training.

Third, there was a gap between the vast amount of data collected and the human resources available to analyze the data. The team’s two established researchers spent their time either attending to urgent demands from the field or supervising the students using project data.

Fourth, despite the success of the CBHI, which is now an autonomous body run by women of the community, the Paniyas are not participating, due to their high levels of social and economic exclusion.

These challenges were overcome through a combination of: 1) creating an environment of methodological rigour and community engagement; 2) having a funding partner who was flexible in adjusting to changing timelines and activities; and 3) above all, having the opportunity to undertake a two-phase project over an eight-year period. This amount of time enabled us to ensure that the research we undertook was rigorous, while allowing us, at the same time, to achieve sustained social change.

## List of abbreviations used

CBHI: community-based health insurance scheme; CDS: Centre for Development Studies; IDRC: International Development Research Centre; LMIC: low and middle-income countries; MDGs: Millenium Development Goals; NGO: non-governmental organizations; PI: primary investigator; SNEHA: Sanjeevani Network of Health Associations; ST: Scheduled Tribes.

## Competing interests

The authors declare that they have no competing interests.

## Authors' contributions

SH and DN were principal investigators of the initiative and KM was a PhD student (in phase 1) and co-researcher (in phase 2) of the initiative. SH, KM, and DN drafted the manuscript. All authors read and approved the final manuscript.
